# Increased IL-33 in Synovial Fluid and Paired Serum Is Associated with Disease Activity and Autoantibodies in Rheumatoid Arthritis

**DOI:** 10.1155/2013/985301

**Published:** 2013-09-09

**Authors:** Sumei Tang, Heqing Huang, Fanlei Hu, Wei Zhou, Jianping Guo, Huirong Jiang, Rong Mu, Zhanguo Li

**Affiliations:** ^1^Department of Rheumatology and Immunology, Peking University People's Hospital, 11 South Xizhimen Street, Beijing 100044, China; ^2^Department of Rheumatology and Immunology, The First Affiliated Hospital of Xiamen University, Xiamen 361003, China; ^3^Department of Rheumatology and Immunology, Peking University First Hospital, 11 South Xizhimen Street, Beijing 100034, China; ^4^Strathclyde Institute of Pharmacy and Biomedical Sciences, University of Strathclyde, Glasgow G40RE, UK

## Abstract

*Objectives*. IL-33, a newly found cytokine which is involved in joint inflammation, could be blocked by a decoy receptor—sST2. The expression and correlation of IL-33 and sST2 in rheumatoid arthritis (RA) are of great interest. *Methods*. Synovial fluid (SF) was obtained from 120 RA and 30 osteoarthritis (OA) patients, and paired sera were collected from 54 of these RA patients. The levels of IL-33 and sST2 were measured by ELISA. *Results*. SF IL-33 was significantly higher in RA than in OA, which was correlated with disease activity score 28, erythrocyte sedimentation rate, rheumatoid factor (RF)-IgM, RF-IgG, glucose phosphate isomerase (GPI), and immunoglobulin. Serum IL-33 was correlated positively with SF IL-33 in RA. Furthermore, it was correlated with RF-IgM and GPI. sST2 was partly detectable in RA (13 out of 54, 24.1%), while not in OA. Serum sST2 in RA had no significant correlation with serum IL-33 or SF IL-33. However, SFs from both RA and OA patients did not express sST2. *Conclusions*. This study supported that IL-33 played an important role in the local pathogenesis of RA. Considering the tight correlation between IL-33 and clinical features, it may become a new target of local treatment.

## 1. Introduction

Rheumatoid arthritis (RA) is a chronic, inflammatory autoimmune disease characterized by the joints erosion and damage. Current research suggests that cytokines play important roles in the immunopathogenesis of RA.

IL-33, a member of the IL-1 cytokine family, is involved in the inflammation of RA via the IL-1 receptor-related protein ST2, and the recruitment of IRAK, IRAK4, MyD88, and TRAF6 to ST2, ultimately leading to the activation of NF-*κ*B and MAP kinases [1]. The soluble form of ST2-sST2, is considered as a decoy receptor to block the effect of IL-33. IL-33 exacerbates the disease severity of collagen-induced arthritis (CIA) which is an animal model of RA disease [2]. Inhibition of IL-33 signaling pathway through blocking anti-ST2 antibody attenuated the severity of CIA [3]. It was reported that sST2 administration could also attenuate the severity of CIA [4]. Some recent studies have further shown that IL-33 is expressed by the synovial fibroblasts from RA patients [2, 3, 5] and the serum level of IL-33 is abnormally elevated in these patients [6–9]. However, the levels of IL-33 and sST2 in the synovial fluid (SF) and whether they are associated with disease activity are less known. 

In this study, we compared the expression of IL-33 and sST2 in SF and paired serum samples of patients with RA to that with osteoarthritis (OA) and analyzed their association with clinical characteristics of RA disease. 

## 2. Materials and Methods

### 2.1. Patients and Samples

SF samples were obtained from 120 patients with RA and 30 patients with OA in an outpatient clinic of the Department of Rheumatology and Immunology, Peking University People's Hospital, from December 2009 to December 2010. Paired sera of 54 RA patients and 12 OA patients were also collected. All patients were grouped according to the revised criteria of the American College of Rheumatology for RA [10] or for OA [11]. The study was approved by the ethics committee of People's Hospital, Peking University, according to the Declaration of Helsinki. All patients had been informed and signed the consent for participation in the study. The details of the patients including gender, age, and disease duration were summarized in Table 1. 

### 2.2. Measurement of IL-33 and sST2

Serum and SF IL-33 concentrations were determined with a commercial ELISA kit (DY3625, R&D Systems, Minneapolis, MN, USA). Serum samples were diluted 1 : 2 in sample dilution buffer. Soluble ST2 was assessed by ELISA using a commercial detection system (DST200, R&D Systems, Minneapolis, MN, USA). The experiments were performed according to the manufacturer's instructions.

### 2.3. Clinical Data and Inflammation Marker Analysis

Clinical data were recorded at the time of sample collection as the following index: age, sex, disease duration, number of swollen joints, and number of tender joints. Erythrocyte sedimentation rate (ESR) was evaluated by the Westergren method. Serum levels of immunoglobulins (IgG, IgM, and IgA,), complements (C3, C4), C-reactive protein (CRP), and rheumatoid factor (RF)-IgM were examined by immunonephelometry method. Antikeratin antibodies (AKA) and antiperinuclear factor (APF) were tested by indirect immunofluorescence assay. Anti-citrullinated peptide (anti-CCP) antibodies, glucose phosphate isomerase (GPI), and RF-IgG were tested by ELISA. The 28-joint count Disease Activity Score (DAS28) was evaluated as described [12].

### 2.4. Statistical Analysis

Data analyses were performed using SPSS 13.0 for Windows. Results are presented as the mean ± SD and percentage. Quantitative data were compared by the Mann-Whitney *U* test. Qualitative data were compared by the Pearson's chi-square test. Paired samples were analyzed using the Wilcoxon matched pairs test. A difference between groups was considered significant if *P* < 0.05. Spearman's rank correlation test was used to assess relationships between two variables. Correlation was considered significant if *P* < 0.05.

## 3. Results

### 3.1. IL-33 in Matched Serum-SF Samples of RA

**Figure 1:**
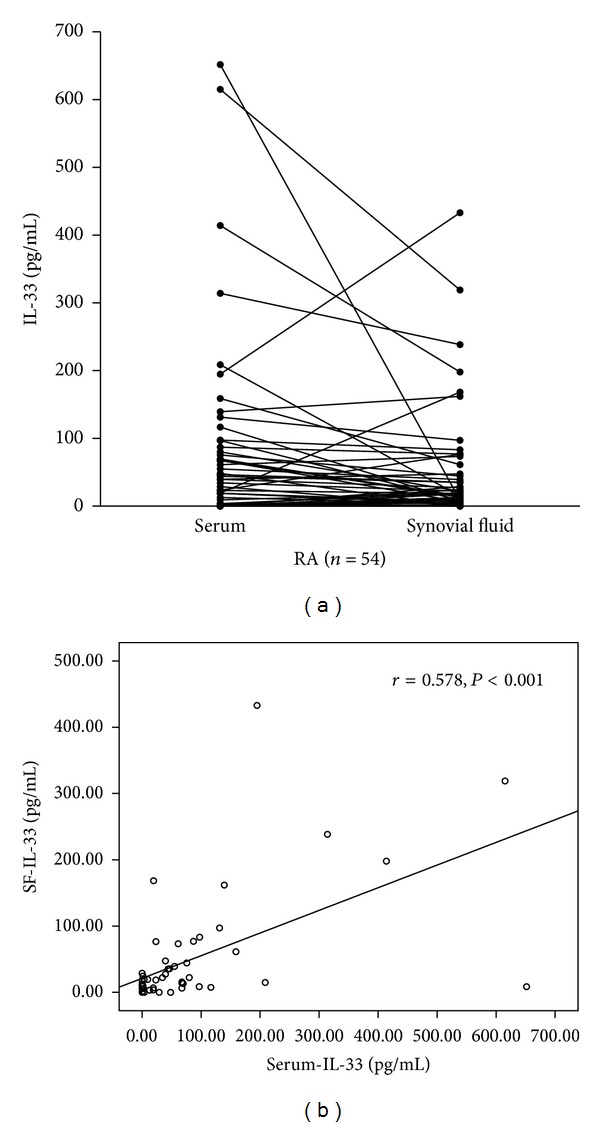
IL-33 levels in serum and SF samples in 54 RA patients were measured simultaneously. The levels in SF (median 15.24 pg/mL) were lower than those in sera (median 31.64 pg/mL (a)). A significant correlation between two groups was estimated by Spearman's correlation test (*r* = 0.578, *P* < 0.001 (b)).

IL-33 level was measured in the matched serum-SF samples of 54 RA patients. The level of IL-33 in SF (median 15.24 pg/mL) was lower than that in sera (median 31.64 pg/mL, Figure 1(a)). Spearman's correlation test was used to analyze the correlation of IL-33 levels in SF and in serum of RA. There is a significant correlation between two groups (*r* = 0.578, *P* < 0.001, Figure 1(b)). 

### 3.2. IL-33 Levels in SF of RA and OA

**Figure 2:**
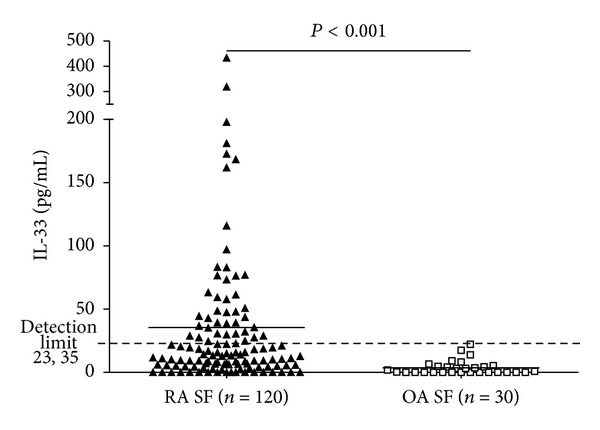
IL-33 levels in SF of RA and OA. SF IL-33 was detectable in 43 of the 120 patients with RA (35.8%), whereas it was nondetectable in patients with OA. The concentration in SF from RA was significantly higher than that from OA (*P* < 0.001).

 The minimal concentration of standard (23.35 pg/mL) was assumed as the detection limit. SF IL-33 was detectable in 43 of the 120 patients with RA (35.8%), while none of the 30 OA patients had detectable level of IL-33 in their SF. SF IL-33 level in RA patients was significantly higher than that of OA patients (35.56 ± 62.56 pg/mL versus 3.66 ± 5.63 pg/mL, *P* < 0.001, Figure 2). 

**Figure 3:**
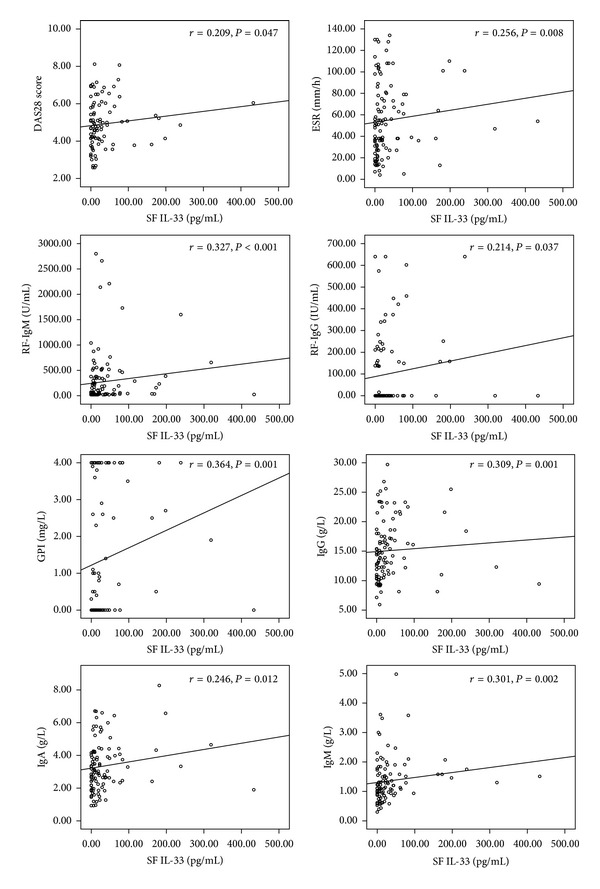
The significant correlations between SF IL-33 and clinical features including disease activity features (ESR, DAS28 Score) and auto-antibodies (RF-IgM, RF-IgG, GPI, IgA, IgG, and IgM). ESR: erythrocyte sedimentation rate; RF: rheumatoid factor; GPI: glucose phosphate isomerase.

A correlation was analyzed between the clinical features and IL-33 levels in SF and serum of patients with RA. Comparing to the serum IL-33, SF IL-33 level in RA had more correlations with clinical features including disease activity features (ESR, DAS28 score) and autoantibodies (RF-IgM, RF-IgG, GPI, IgA, IgG, and IgM). The data was shown in Table 2 and Figure 3.

### 3.3. SF IL-33 Was Correlated with Disease Activity in RA

**Figure 4:**
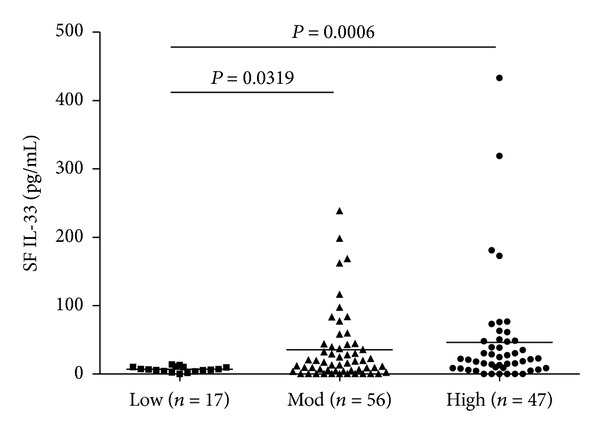
Interleukin 33 (IL-33) levels in synovial fluid (SF) from RA patients. RA patients were categorized into 3 groups according to the Disease Activity Score based on ESR. Low: low activity group; Mod: moderate activity group; High: high activity group. Symbols represent individual samples. Horizontal bars represent median IL-33 levels.

These 120 patients with RA were classified into 3 groups according to the DAS28-ESR: the high activity group (47 patients) was defined as DAS28-ESR > 5.1; the moderate activity group (56 patients) was defined as 5.1 ≥ DAS28-ESR > 3.2; the low activity group (17 patients) was defined as DAS28-ESR ≤ 3.2 [12]. SF IL-33 levels were significantly higher in the high and moderate activity groups than in the low activity group (*P* = 0.0319 and 0.0006, resp.; Figure 4).

Correspondingly, RA patients were divided into SF-IL-33-positive and SF-IL-33-negative groups according to the minimal detection limit of IL-33 (23.35 pg/mL), and the characteristics of these groups are shown in Table 3. It showed that the SF-IL-33-positive group had higher DAS28, ESR, and CRP than the SF-IL-33-negative group.

### 3.4. SF-IL-33 Was Correlated with Autoantibody Production in RA

It was shown in Table 2 that the level of SF-IL-33 was correlated with the levels of several auto-antibodies in serum including RF-IgM, RF-IgG, GPI, IgG, IgA, and IgM in patients with RA. 

We also found in Table 3 that the SF-IL-33-positive group had higher levels of RF-IgM, IgG, IgA, and IgM than the SF-IL-33-negative group. The positive frequency of GPI was also higher in the SF-IL-33-positive group than in the SF-IL-33-negative group. 

Similarly, SF IL-33 concentration in GPI-positive patients (53.96 ± 71.43 pg/mL) was higher than that in GPI-negative patients (24.85 ± 66.81 pg/mL, *P* = 0.002). A similar trend was identified in RF-IgM-positive patients (42.58 ± 59.85 pg/mL versus 22.98 ± 65.96 pg/mL, *P* = 0.001).

### 3.5. sST2 in Matched Samples of RA and OA

Soluble ST2 concentration in matched serum-SF samples of 54 RA patients and 12 OA patients was also measured by ELISA. The reference serum interval of kit (6.74–20.4 ng/mL) was used as the boundary. It showed that serum sST2 was elevated in 13 of the 54 patients with RA (24.1%) while none of the OA patients had a detectable level of sST2. No significant difference of SF sST2 concentrations was observed between RA (3.14 ± 1.94 ng/mL) and OA (3.20 ± 1.71 ng/mL) patients. Furthermore, there was no correlation between serum sST2 and serum IL-33 level or SF IL-33 level, showing that the relationship between sST2 and IL-33 in RA needs more effort to be understood.

## 4. Discussion

According to our knowledge, this is the largest study of IL-33 and sST2 in SF and matched serum of RA and OA patients. Comparing to the former studies [6–9], it could provide the more believable results about the expression of IL-33 in RA. Based on the present study, we found that there was an obvious difference of IL-33 levels between SF and matched serum. We also demonstrated that SF-IL-33 in RA patients had higher levels than OA patients, whether with positive rate or mean expression. Additionally, our study showed that there was a positive correlation between SF-IL-33 and disease activity or RA-associated auto-antibodies, which supported the hypothesis that IL-33 plays an important role in the pathogenesis of RA. 

In our study, SF IL-33 levels were significantly higher in the high and moderate activity groups than in the low activity group. On the other hand, the SF-IL-33-positive group had higher DAS28, ESR, and CRP than the SF-IL-33-negative group. It suggested that SF IL-33 was closely associated with systemic inflammation. 

Furthermore, the SF-IL-33-positive group had higher levels of RF-IgM, IgG, and IgA and positive frequency of GPI than the SF-IL-33-negative group. The result of our former study [7], that serum IL-33 is associated with antibody production, was also proved with SF-IL-33. All these antibodies are important features for diagnosis and prognosis [13–15], so we considered that IL-33 may be a risk factor for poor prognosis in RA. Further study is deserved about the mechanism of IL-33 in inducing antibody production.

As a specific ligand of IL-33, sST2 can serve as an anti-inflammatory mediator through binding with IL-33, sequentially decrease the interaction of IL-33 and ST2L [16]. Therefore, we need to know the expression of sST2 in RA SF and serum to further understand its function in the pathogenesis. In our study, the positive frequency of serum sST2 in RA patients was higher than that in OA patients, but there was no significant difference. The concentrations of SF sST2 also had no difference between RA and OA. Since the expression of sST2 could be induced in various mesenchymal and hematopoietic cells by a lot of stimuli [17–20], therefore, it is possible that the inflammatory milieu is responsible for the level of sST2 in OA. We tried to find out the relationship between serum sST2 and serum IL-33 or the other clinical features in RA, but no correlation was observed. The similar result was also shown in a previous study [9], which was held by a relative low number of samples.

In conclusion, SF IL-33 levels that increased in RA patients could be treated as a sensitive marker of disease activity and were associated with the production of antibodies. The correlated expression of IL-33 between SF and serum suggests that IL-33 plays an important role in the local pathogenesis of RA. It may become a new target of local treatment. The role of sST2 and the therapeutic significance of IL-33/sST2 system in RA need further research.

## Figures and Tables

**Table 1:** Characteristics of enrolled patients with RA and OA.

Samples	Diseases	*n *	Gender		Age		Disease duration	
			M/F	*P *	Range (year, mean ± SD)	*P *	Range (year, mean ± SD)	*P *
SF	RA	120	23/97	0.457	12–81 (53.8 ± 16.1)	0.111	0.3–30 (9.4 ± 8.4)	0.847
	OA	30	4/26		40–77 (57.8 ± 11.3)		0.1–30 (11.1 ± 8.4)	

Serum	RA	54	6/48	0.347	23–81 (55.6 ± 13.4)	0.423	0.3–30 (8.2 ± 7.4)	0.524
	OA	12	3/9		53–72 (61.4 ± 10.4)		0.1–30 (6.6 ± 8.2)	

RA: rheumatoid arthritis; OA: osteoarthritis; SF: synovial fluid.

**Table 2:** Correlation analysis between SF-serum IL-33 (pg/mL) and clinical and laboratory variables.

Measurements	SF IL-33 (*n* = 120)		Serum IL-33 (*n* = 54)	
	*r *	*P *	*r *	*P *
Age (yrs)	−0.106	0.257	−0.014	0.921
Tender joint count, 0–46 joints	0.095	0.347	0.327	0.020*
Swollen joint count, 0–48 joints	0.192	0.056	0.134	0.354
DAS28-ESR	0.209	0.047*	0.253	0.102
ESR (mm/h)	0.256	0.008*	0.213	0.150
CRP (mg/L)	0.166	0.073	0.219	0.116
C3 (g/L)	0.113	0.260	0.267	0.061
C4 (g/L)	0.039	0.698	0.180	0.211
IgA (g/L)	0.246	0.012*	0.107	0.457
IgG (g/L)	0.309	0.001*	0.136	0.343
IgM (g/L)	0.301	0.002*	0.220	0.121
RF-IgM (U/mL)	0.327	<0.001*	0.342	0.011*
RF-IgG (IU/mL)	0.214	0.037*	0.183	0.208
GPI (mg/L)	0.364	0.001*	0.505	0.000*
Anti-CCP (RU/mL)	0.104	0.284	0.052	0.716

*P < 0.05. Spearman's correlation test was used.

**Table 3:** The comparison of clinical features between SF-IL-33-positive and SF-IL-33-negative groups.

Measurements	SF-IL-33-positive (*n* = 43)	SF-IL-33-negative (*n* = 77)	*Z* (*x* ^2^)	*P *
Age (yrs)	49.9 ± 18.5	56.0 ± 14.2	−1.490	0.136
Gender (M/F)	11/32	12/65	1.220	0.325
Disease duration (yrs)	10.2 ± 8.9	9.0 ± 8.0	−0.515	0.607
Tender joint count	8.1 ± 8.3	6.9 ± 7.8	−0.847	0.397
Swollen joint count	8.1 ± 8.0	5.4 ± 7.0	−1.789	0.074
DAS28	5.3 ± 1.2	4.7 ± 1.3	−2.103	0.035*
ESR (mm/h)	66.7 ± 33.0	48.7 ± 33.2	−2.976	0.003*
CRP (mg/L)	49.3 ± 39.4	34.9 ± 32.3	−2.055	0.040*
RF-IgM (U/mL)	440.7 ± 646.0	186.8 ± 378.6	−3.564	<0.001*
RF-IgG-positive	46.5% (20)	27.3% (21)	3.136	0.110
GPI-positive	67.4% (29)	41.6% (32)	5.332	0.026*
IgA (g/L)	3.9 ± 1.6	3.1 ± 1.4	−2.258	0.024*
IgG (g/L)	17.1 ± 5.4	14.0 ± 4.7	−2.888	0.004*
IgM (g/L)	1.6 ± 0.8	1.2 ± 0.7	−3.064	0.002*
C3 (g/L)	1.2 ± 0.4	1.1 ± 0.3	−1.002	0.317
C4 (g/L)	0.2 ± 0.1	0.2 ± 0.1	−0.077	0.939
Anti-CCP (RU/mL)	107.9 ± 80.3	97.0 ± 81.6	−0.937	0.349
AKA-positive	21 (48.8%)	42 (54.5%)	0.269	0.383
APF-positive	25 (58.1%)	50 (64.9%)	0.367	0.651

*P < 0.05.

SF IL-33-positive: SF IL-33 ≥ 23.35 pg/mL; SF IL-33-negative: SF IL-33 < 23.35 pg/mL.
